# Kleefstra Syndrome—Dental Manifestations and Needs: A Case Report with a Literature Review

**DOI:** 10.1155/2023/2478465

**Published:** 2023-09-22

**Authors:** Victoria Karlak, Jakub Jankowski, Julia Kolasińska, Kacper Nijakowski

**Affiliations:** ^1^University Centre of Dentistry and Specialised Medicine, 60-812 Poznan, Poland; ^2^Student's Scientific Group in Department of Conservative Dentistry and Endodontics, Poznan University of Medical Sciences, 60-812 Poznan, Poland; ^3^Department of Conservative Dentistry and Endodontics, Poznan University of Medical Sciences, 60-812 Poznan, Poland

## Abstract

Kleefstra syndrome (KS) is a rare genetic disorder (prevalence < 1/1 000 000) characterised by autistic spectrum disorder (ASD), childhood hypotonia, and seizures. A typical facial appearance includes microcephaly, arched eyebrows, synophrism, hypertelorism, a short nose, midface hypoplasia, prognathism, and tongue protrusion. This case report presents a 19-year-old female with KS from a dentist's perspective. The patient demonstrates limited mouth opening with a slight deviation of the mandible on the left side. The mandibular prognathism and anterior open bite are visible. A generalised inflammatory gingival enlargement is most likely a response to local irritants like plaque and calculus but is enhanced by the patient's mouth breathing habit. Also, the tongue is unusually large—macroglossia. Dental anomalies were diagnosed by digital panoramic radiograph, including dilaceration of the maxillary left second premolar and taurodontism. The patient was qualified to be treated under general anaesthesia with multiple extractions, restorations, hygienisation procedures, and tooth remineralisation. This individual should also be considered for orthodontic treatment and an eventual tongue reduction procedure. In the case of KS, multidisciplinary cooperation as well as frequent dental check-ups with fluoride prophylaxis are recommended. Unfortunately, dental treatment is still not an integral part of taking care of disabled children and youth with rare diseases.

## 1. Introduction

Kleefstra syndrome (KS) is a rare genetic disorder (prevalence < 1/1 000 000) characterised by autistic spectrum disorder (ASD), childhood hypotonia, and seizures. KS has a clinically recognisable phenotype mainly caused by a heterozygous 9q34.3 deletion in the *EHMT1* gene. A typical facial appearance includes microcephaly, arched eyebrows, synophrism, hypertelorism, a short nose, midface hypoplasia, prognathism, and tongue protrusion. Its management requires multidisciplinary cooperation. Differential diagnosis should consider such genetic disorders as Down syndrome, Smith-Magenis syndrome, Pitt-Hopkins syndrome, or Angelman syndrome [1].

Following the initial diagnosis, several evaluations of a proband with KS are recommended, including one to assess dental issues. This case report presents a 19-year-old female with KS from a dentist's perspective.

## 2. Case Presentation

Proband was diagnosed with KS at age 17. Born in 2004 to healthy nonconsanguineous parents, a girl was delivered on time with a birth weight and height average (25^th^-50^th^ percentiles) after an uneventful pregnancy. Immediately after birth, the APGAR score was 8 because of colour and muscle tone. The newborn had three subdural hematomas and was overseen by a neurologist at the maternity ward, where she stayed for five days. Both mother and newborn were discharged from the hospital as healthy.

The newborn was suspiciously calm and hypotonic but did not have a feeding or breathing disorder. Her paediatrician did not order any testing because her weight and height were normal during the baby examination from 0 to 12 months.

Around age one, because her developmental milestones were delayed and she was still hypotonic, the mother consulted another paediatrician. The new physician immediately suspected a genetic disorder and referred the patient to a genetic diagnostic centre and to physical therapy. Due to financial situation, the child had only three genetic tests done throughout the 15 years, which allowed him to exclude Down syndrome at first, Prader-Willi syndrome at second, and finally lead to a Kleefstra syndrome diagnosis. The comparative genomic hybridisation confirmed the genomic imbalance involving a ∼503 kb terminal deletion of the long arm of chromosome 9 in region 9q34.3 (*EHMT1* and *CACNA1B* genes) and a ∼911 kb terminal duplication of the short arm of chromosome 3 in region 3p26.3 (*CHL1* gene).

**Figure 1:**
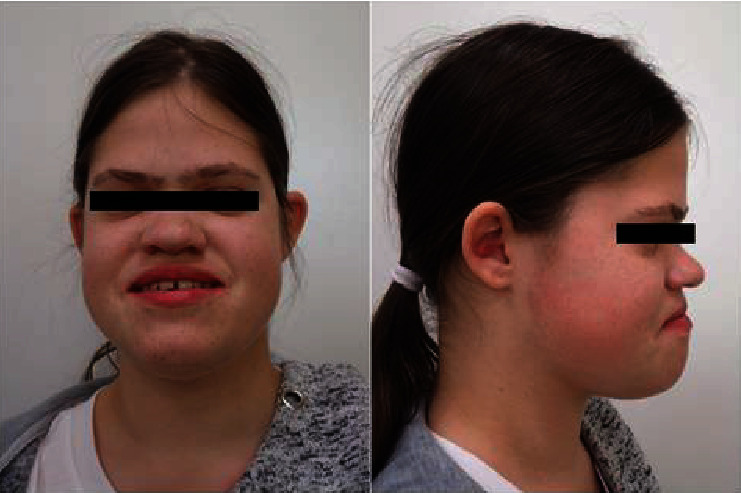
Patient's profiles *en face* and side.

Comparing one side of the head and neck to the other, no significant asymmetries were noted (Figure 1). The lymph nodes in the head and neck area were either not palpable or mobile and nontender. A basic temporomandibular joint examination was done, and no clicking or crepitus was noted. The patient presents a limited mouth opening with a slight deviation of the mandible on the left side. Palpation of the muscles of mastication may indicate that the patient suffers from bruxism.

The apparent height of the lower lip vermilion is excessive, and the upper lip vermilion is cupid bow-shaped. The labial mucosa is unaltered. The buccal and vestibular mucosas are smooth, moist, and shiny. Hard and soft palate with no visible abnormalities. Between fauces, there are no visible tonsils because they have been surgically removed during childhood. The posterior pharynx wall and the uvula are commonly seen as lymphoid tissue. The tongue is unusually large—macroglossia. The oral portion of the tongue is moist and pink, with visible filiform, fungiform, and circumvallate papillae slightly coated with dental biofilm. The floor of the mouth appears moist and very vascular. The normal anatomy of the area was identified, including sublingual caruncles and sublingual folds. The lingual frenulum and the inferior labial frenulum are absent. The superior labial frenulum is causing a large gap between the upper two front teeth. By observing the saliva on the floor of the mouth, we classified the consistency of the saliva as sticky.

**Figure 2:**
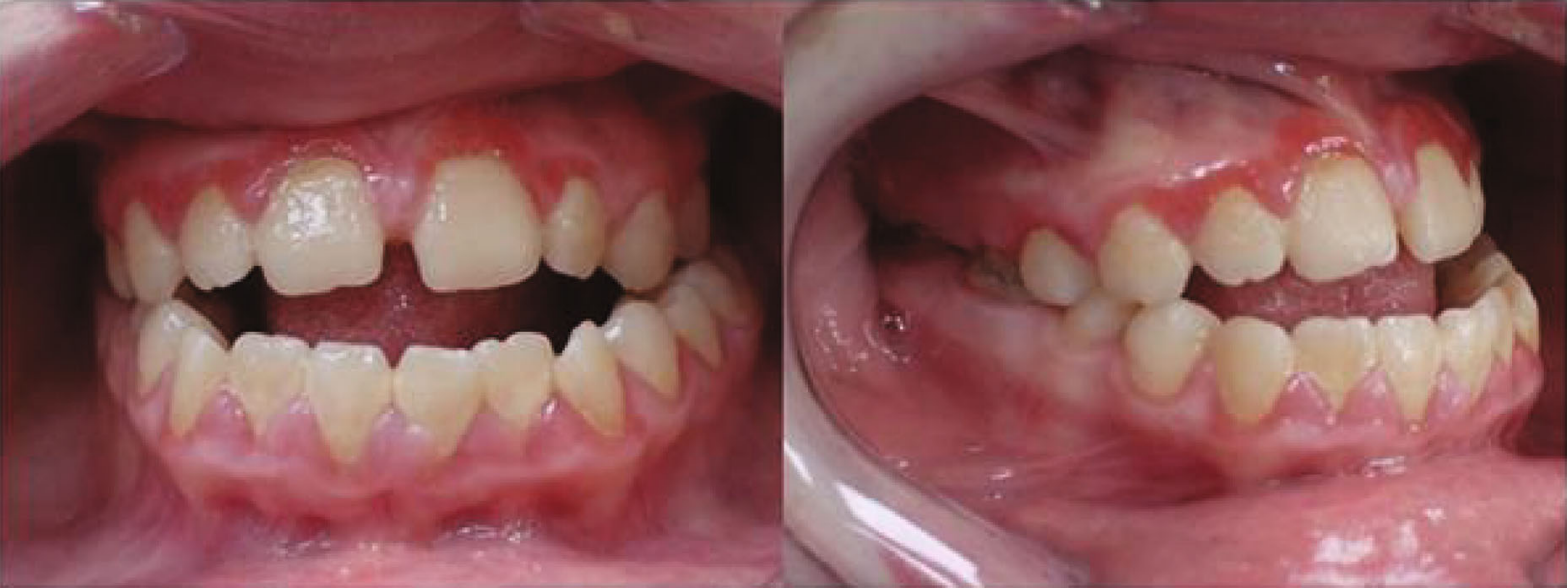
Intraoral bite photographs on the front and side.

**Figure 3:**
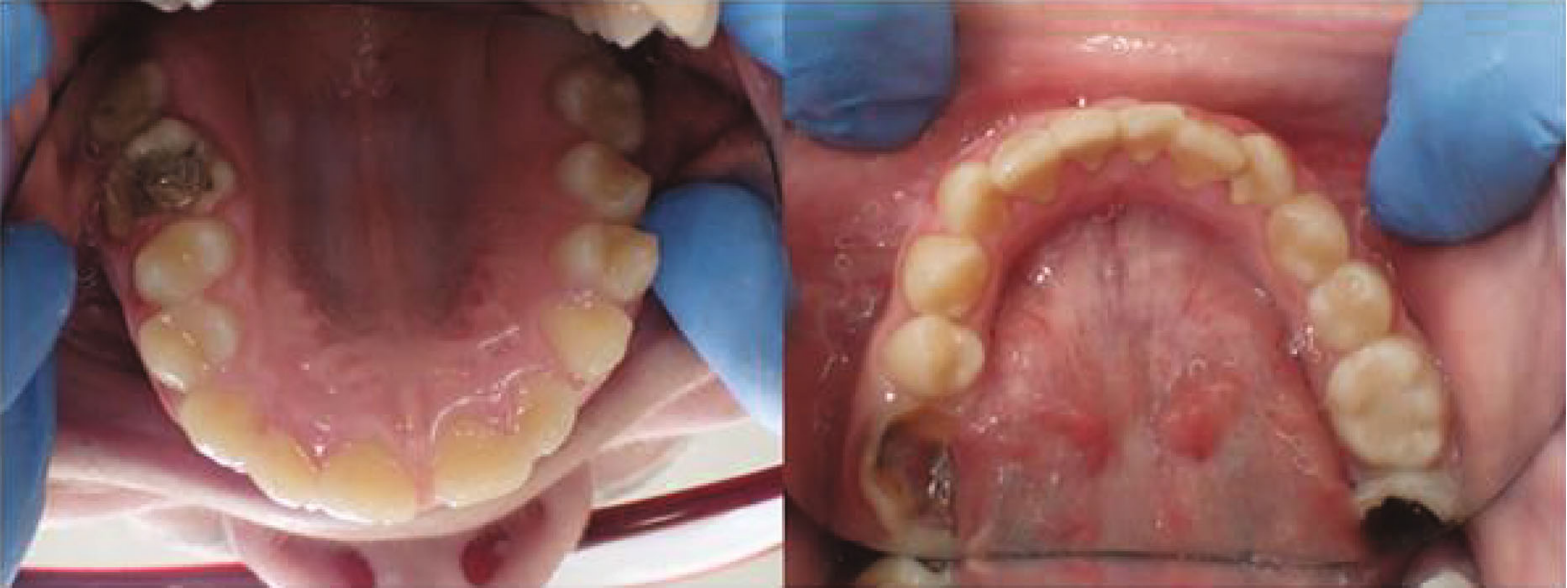
Intraoral photographs of maxillary and mandibular dental arches.

Anterior open bite malocclusion is visible, as is mandibular prognathism (Figure 2). A generalised inflammatory gingival enlargement is most likely a response to local irritants like microbial deposits (plaque and calculus) but is enhanced by the patient's mouth breathing habit (Figure 3).

Noticing heavy plaque, the degree of gingivitis was assessed using the gingival index (according to Löe and Silness [2]), scoring “2” as moderate. During the intraoral examination, using International Caries Detection and Assessment System II (ICDAS II), three mandibular molars and one maxillary molar were coded “6” because of extensive decay cavities. The maxillary left first molar was previously removed.

**Figure 4:**
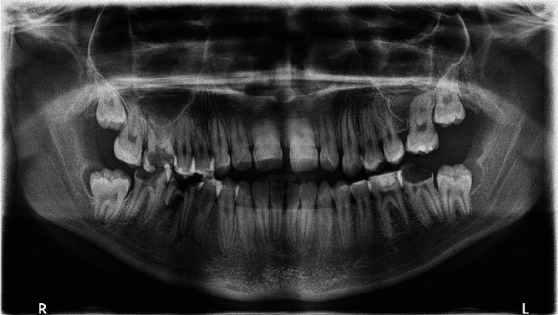
Digital panoramic radiograph of the patient.

**Figure 5:**
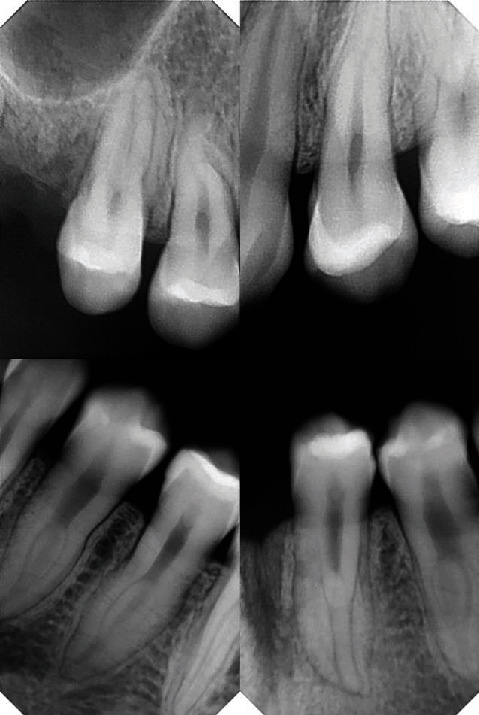
Digital periapical radiographs of the patient's premolars.

Dental anomalies, including root anomalies, were diagnosed by digital panoramic and periapical radiographs (Figures 4 and 5). The maxillary right first and left second premolars most seemed to demonstrate lacerations. All other premolars manifested root canal systems analogous to molars with taurodontism features and supernumerary roots. Also, root resorption of the first mandibular molars was found.

The patient was qualified to be treated under general anaesthesia with multiple extractions, restorations, teeth cleaning, and tooth remineralisation. This individual should be considered for orthodontic treatment and an eventual tongue reduction procedure.

Dental procedures carried out under general anaesthesia (narcosis) are performed by two teams: dental and anaesthesia. The anaesthesia team was responsible for safely putting the patient under anaesthesia by administering drugs intravenously, monitoring her throughout the procedure, waking her after the surgery, and discharging her on the same day, based on the anaesthesiologist's judgment.

The dental team performed a dental examination. Caries lesions in both maxillary first premolars and mandibular right second premolar were diagnosed by only inspection and probing and restored with glass-ionomer and composite fillings. Debridement with an ultrasonic scaler and hand-held instruments was performed.

Unrestorable remaining first molars and mandibular second molars were extracted. Absorbable sutures and dental collagen sponges were applied to the extraction wounds. The extraction sites were each infiltrated with 1 ml of local anaesthetic (articaine + epinephrine). Because the right maxillary molar on the dental panoramic radiograph looked close to the lowest point of the antral floor, a local buccal flap was allowed to close the possible oroantral fistula.

Fluoride varnishes were applied, increasing the fluoride content in the enamel and preventing caries. The operation took 55 minutes. It was advised to take regular pain-relieving medicine for the first 24 to 48 hours: paracetamol and/or ibuprofen at the dosages recommended on the pack. The patient (mental age around 10 years old) and especially her guardians were given advice on proper oral hygiene.

Full-mouth rehabilitation under general anaesthesia in daycare services is a safe and effective way of providing dental care for people with genetic disorders such as KS.

## 3. Discussion

To the best of our knowledge, the case of our patient is the second KS report described in Poland. Although dozens of cases have been published around the world, ours is the first to report an accurate picture of the oral health status along with radiological documentation presenting anomalies in the structure of the tooth roots. So far, the presented cases have focused on the description of the outermost appearance, taking into account the profile caused by midface hypoplasia resulting in the alleged forebite. In addition, we proposed a prophylaxis and treatment plan for patients with KS syndrome.

For the literature review, we used the advanced search builder in the PubMed database with the search query “(Kleefstra syndrome) AND (dental OR facial OR orthodontic OR oral)”. Out of the 32 papers selected by 28 May 2023, we selected 11 clinical case papers and one observational study presenting a series of cases that presented orofacial symptoms. Additionally, we included two eligible papers found in Google Scholar (the first Polish case report and the first cohort of 12 patients with subtelomeric deletions of chromosome 9q34).

The results collected in Table 1 present 18 patients with KS with different types of mutations (data are arranged chronologically). Female paediatric patients predominated among the included case reports (13 vs. 5). These case reports came mainly from Europe and the USA, but only one came from Asia (Iran). The authors reported most commonly microdeletions in the 9q34.3 region involving *EHMT1*, but also mutations in other genes such as *KMT2C*. The interesting observation was the familial occurrence of this syndrome, where the proband's mother was the mosaic carrier of the 9q34.3 deletion.

In patients with KS, highly recognisable orofacial anomalies were midface hypoplasia (*n* = 10), palpebral fissures (*n* = 8), hypertelorism (*n* = 8), ear anomalies (*n* = 8), and tented or everted lips (*n* = 6). Less common were symptoms such as protruding tongue, which occurred in a Polish 9-year-old girl (with terminal deletion of the long arm of chromosome 9 in band 9q34.3), a 3-year-old girl (with 145 kb intragenic duplication in the *EHMT1* gene), and a 14-month-old boy (with 46 kb 9q34.3 deletion involving the *EHMT1* gene) who also developed synophrys, reported additionally in two other patients: a 3-year-old girl (with a microdeletion in the 9q34.3 region in the *EHMT1* gene) and a 16-year-old girl (with the novel c.2426C>T (p.P809L) missense variant in *EHMT1*).

The study by Willemsen et al. [15] included 16 patients (8 males/8 females) with a 9q34.3 deletion and 11 patients (3 males/8 females) with an intragenic *EHMT1* mutation. In patients with a 9q34.3 deletion, highly recognisable facial features comprised midface hypoplasia, hypertelorism, prominent eyebrows, prognathism, and a cupid bow or tented upper lip and everted lower lip. Similar observations were reported in patients with an intragenic *EHMT1* mutation. In turn, eye anomalies were reported in 3 patients: 10- and 18-year-old boys with a 9q34.3 deletion and a 9-year-old girl with an intragenic *EHMT1* mutation. Also, only two patients demonstrated tooth anomalies: an 11-year-old girl with 9q34.3 deletion and a 2-year-old boy with an intragenic *EHMT1* mutation.

Moreover, Stewart et al. [16] described 19 patients with submicroscopic subtelomeric deletions of the long arm of chromosome 9: 12 own patients (5 males/7 females) and 7 patients previously reported (all males). In cytogenetic findings, the vast majority was .ish del(9)(qtel-), especially in males.

Among craniofacial abnormalities, microcephaly dominated (16/19 patients), and brachycephaly cooccurred in only 8/17. Other facial changes included frontal bossing, midface hypoplasia, and coarse facies. In turn, ear and eye muscle changes were not widespread among these patients. Nasal defects, such as a short nose, anteverted nares, and broad nasal root, as well as synophrys, were present in more than half of the respondents, which makes them the most common facial defects in patients with subtelomeric deletions of chromosome 9q. Among the ear defects, low-set ears (5/19) dominated next to overfolded helices (4/19).

Also, mouth changes were seen in most patients and included a thin or tented upper lip (12/19). A large protruding tongue was observed in 7 patients. In contrast, wide mouth only occurs in 4 patients. High arched palate was reported in both patients with del(9)(q34.3) and two patients with .ish del(9)(qtel-) (4/19)—male gender predilection dominated here (3 : 1). In addition to the defects previously described, some male patients presented prognathism, facial asymmetry, and widely spaced teeth. Interestingly, the rare finding of neonatal teeth was observed in three patients with cytogenetically visible deletions, as was emphasised in the individual with the largest deletion in this cohort.

## 4. Conclusions

In this case of Kleefstra syndrome, multidisciplinary cooperation, as well as frequent dental check-ups with fluoride prophylaxis, is recommended. Unfortunately, dental treatment is still not an integral part of taking care of disabled children and youth with rare diseases.

## Figures and Tables

**Table 1:** Included case reports demonstrating orofacial findings in Kleefstra syndrome.

Case report	Setting	Age	Gender	Mutation	Orofacial findings
Willemsen et al. [3]	The Netherlands	5 y	F	∼200 kb deletion in chromosome band 9q34.3	Hypertelorism, broad and depressed bridge of the nose, flared nostrils, mild midface hypoplasia, and broad mouth with full lips
		NR (proband's mother)	F	Mosaic carrier of 9q34.3 deletion	Hypoplastic midface, small palpebral fissures, depressed nasal bridge, and anteverted nares

Campbell et al. [4]	USA	5 d	F	2.1 Mb deletion at 9q34.3 involving 30 *OMIM* genes, including *EHMT1* and *NOTCH1*	Periorbital fullness, hyperteloric appearance with long, downslanting palpebral fissures, prominent earlobes, and overfolded helices

Schwaibold et al. [5]	Germany	3 y	F	145 kb intragenic duplication in *EHMT1* gene	Square, brachycephalic face with prominent forehead and frontal bossing, slight midface hypoplasia, hypertelorism with mildly downslanting palpebral fissures, synophrys, small nose with anteverted nostrils and deep-set nasal root, mild prognathism, deep-set posterior rotated ears, full cheeks and prominent philtrum; mouth held mostly opened with cupid bowed upper lip, full lower lip and slightly protruding tongue

Blackburn et al. [6]	USA	4 y	F	Novel c.2426C>T (p.P809L) missense variant in *EHMT1*	Distinctive craniofacial features, coarse facies, and strabismus
		16 y	F		Microbrachycephaly, coarse facies, hypertelorism, synophrys, and prognathia/midface hypoplasia

Hadzsiev et al. [7]	Hungary	22 m	F	9q34.3 terminal deletion (2.188 Mb)	Brachycephaly, flat face, midface hypoplasia, downslanting palpebral fissure, convergent strabismus, short nose, high palate, tented lip, and severe generalised hypotonia
		30 m	M	1.211 Mb deletion in 9q subtelomeric region	Brachymicrocephaly, flat face, midface hypoplasia, hypertelorism, short nose, tented lip, thick lower lip, pointed chin, malformed ears, and mild hypotonia

Blackburn et al. [8]	USA	18 y	F	Novel de novo single-base frameshift deletion in *EHMT1* (Chr9(GRCh37): g.140637927_140637928del; NM_024757.4(EHMT1): c.928_929del; NP_079033.4: p.Arg310Aspfs^∗^4)	Microbrachycephaly, ear crease, widely spaced eyes, epicanthal folds, upslanted palpebral fissures, and mild intermittent esotropia

Noruzinia et al. [9]	Iran	3 y	F	Microdeletion in the 9q34.3 region, haploinsufficiency, and LOH in *EHMT1* gene	Flat face, midface hypoplasia, coarse facies, synophrys, upslanting palpebral fissures, and anteverted nostrils

Ciaccio et al. [10]	Italy	14 m	M	46 kb 9q34.3 deletion involving *EHMT1* (exons 24–27)	Mildly broad forehead, synophrys, midface hypoplasia, depressed nasal bridge, short nose, long and flat philtrum, tented upper lip, everted lower lip, protruding tongue with open carp mouth, low-set ears with upturned ear lobes, and prominent antitragus
		19 m	M	448 kb deletion in 9q34.3 region involving *EHMT1* and *CACNA1B* genes	Broad forehead, widely spaced eyes, epicanthal folds, upslanting palpebral fissures, midface hypoplasia, short nose, anteverted nares, depressed nasal bridge, low-set ears with prominent ear lobes and thick helix, teeth small, and spaced apart

Okur et al. [11]	USA	18 m	M	Prenatal 9q34.3 microdeletion	Microcephaly, hypotonia, hypertelorism, epicanthal folds, broad nasal bridge, and anteverted nares
		7 m	F	2.9 Mb deletion at 9q34.3	Congenital microcephaly and marked hypotonia
		3 y	F	1.24 Mb deletion of 9q34.3 and 13.24 Mb duplication of Xp22.2 translocated onto 9q34.3	Hypotonia, mild hypertelorism, slightly downslanting palpebral fissures, slightly anteverted nares, and midline sublingual cystic mass

Torga et al. [12]	USA	11 y	M	Heterozygous de novo variant c.2712+1G>A in *EHMT1* gene	Prominent eyebrow, low-set ears, midfacial retrusion, and mild prognathism

Kukla et al. [13]	Poland	9 y	F	Terminal deletion of the long arm of chromosome 9 in band 9q34.3	Brachycephaly, midface hypoplasia, hypertelorism, arched eyebrows, short nose with anteverted nares, open mouth with fleshy everted lower lips, large tongue and thickened ear helices, and dental excrescences in the mandible (when she was born)

Siano et al. [14]	Italy	6 y	F	Novel *KMT2C* missense variant: c.9244C>T	Macrocephalic facies, broad and rounded forehead, hypertelorism, and nose with saddle bridge and bulbous tip

Legend: F, female; M, male; USA, the United States of America; NR, not reported.

## Data Availability

Data are available on request from the corresponding author.
